# A >30-cm circumferential rectal laterally spreading tumor treated with multitunnel endoscopic submucosal dissection and suturing

**DOI:** 10.1016/j.vgie.2025.08.006

**Published:** 2025-09-01

**Authors:** Fatih Aslan, Orhun Cig Taskin, Serhat Ozer, Mete Manici

**Affiliations:** 1 Anesthesiology and Reanimation, Koc University Hospital, Istanbul, Turkey; 2 Pathology, Koc University Hospital, Istanbul, Turkey; 3 Gastroenterology and Advanced Endoscopy, Koc University Hospital, Istanbul, Turkey

## Abstract

**Background and Aims:**

Laterally spreading tumors (LSTs) located in the colon and rectum are lesions with malignant potential and present significant diagnostic and therapeutic challenges, particularly when they reach large sizes. In this case, we present the treatment of a >30-cm circumferential rectal LST using multitunnel endoscopic submucosal dissection (ESD) combined with suturing.

**Methods:**

A 51-year-old female patient underwent colonoscopic evaluation, which revealed a granular-type LST extending circumferentially from the fourth centimeter of the rectum to approximately 15 cm proximally. With the patient under general anesthesia, ESD was performed using the multitunnel technique, followed by full-thickness endoscopic suturing. The procedure was successfully completed.

**Results:**

The lesion was resected en bloc (324 × 214 mm). Histopathologic examination revealed a tubulovillous adenoma with high-grade dysplasia and clear resection margins. The patient was discharged without any reported adverse events. No recurrence or stricture was observed during 24 months of follow-up.

**Conclusions:**

The multitunnel ESD and full-thickness suturing technique is an effective, safe, and function-preserving approach for large circumferential rectal LSTs. We believe that this strategy contributes to both curative resection and the prevention of adverse events such as stricture and bleeding.

## Introduction

Laterally spreading tumors (LSTs) located in the colon and rectum with varying morphologic subtypes carry a certain risk of malignancy, depending on location and surface features and thereby requiring definitive treatment. Various endoscopic approaches have been available for management. Wide-field EMR is a commonly used technique for the treatment of premalignant lesions but carries a notable risk of recurrence.^1^^,^^2^ Endoscopic submucosal dissection (ESD), in contrast, offers the advantage of accurate histopathologic diagnosis, staging, and curative resection. However, depending on the lesion's characteristics, location, and size, ESD has significant preoperative, intraoperative, and postoperative challenges.^3^^,^^4^ This article presents the successful treatment of a giant circumferential rectal LST using a strategic approach that combines the multitunneling technique (MTT) ESD with endoscopic suturing.

## Case report

**Figure 1:**
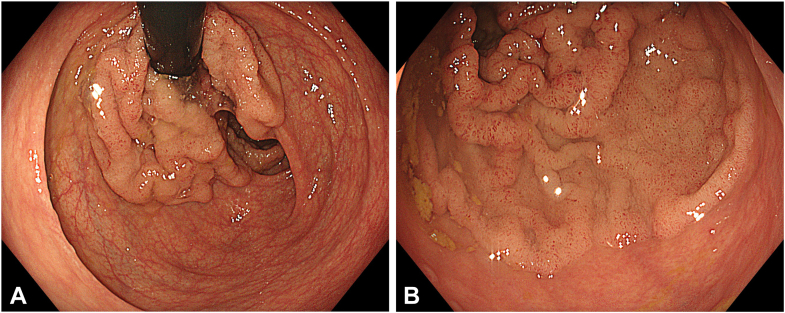
**A,** Endoscopic view of the laterally spreading tumor in retroflexed position. **B,** Endoscopic view of the laterally spreading tumor in straight position.

**Figure 2:**
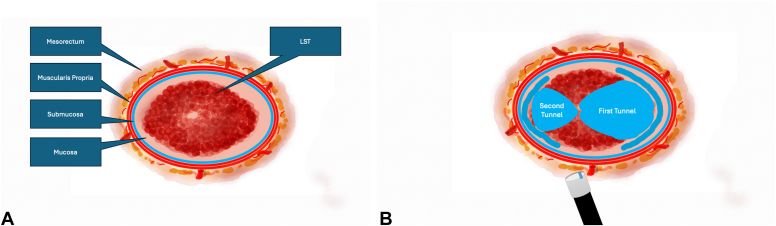
**A,** Schematic view of the laterally spreading tumor (LST) and its location. **B,** Schematic view of the multitunnel technique.

A 51-year-old woman presenting with diarrhea comprising mucous and iron deficiency anemia underwent endoscopic evaluation, which revealed a granular-type circumferential LST extending from the fourth centimeter of the rectum up to approximately 15 cm proximally (Fig. 1A and B). Evaluation with white-light imaging, texture- and color-enhancement imaging, narrow-band imaging, and extended depth of field demonstrated a regular surface and vascular pattern, classified as Japan Narrow-Band Imaging Expert Team type 2a, Paris type 2a, and Kudo type IV. Previous biopsy samples were consistent with tubulovillous adenoma. Endoscopic ultrasonography and magnetic resonance imaging findings revealed neither distant metastasis nor lymphadenopathy. With the patient under general anesthesia, MTT ESD (Fig. 2A and B) and endoscopic suturing were planned. Prophylactic cefazolin sodium (2 g/day) was initiated and continued until discharge.

**Figure 3:**
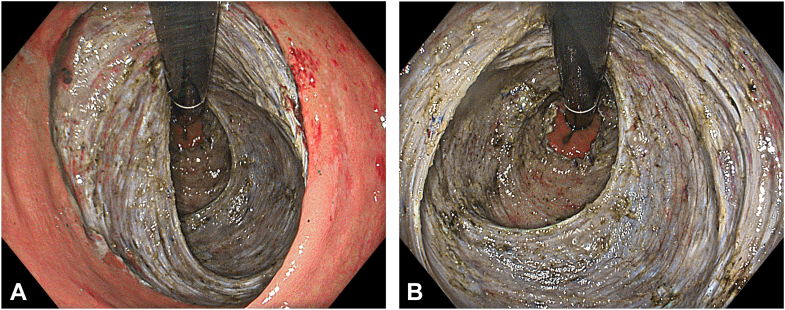
**A,** Endoscopic view of the resection area in retroflexed position under texture- and color-enhancement imaging light mode after endoscopic submucosal dissection. **B,** Endoscopic view of the resection area in retroflexed position under texture- and color-enhancement imaging light mode following endoscopic submucosal dissection.

An endoscopic hood (Olympus, Tokyo, Japan) was attached to a gastroscope tip. Submucosal injection with a solution containing 6% hydroxyethyl starch and indigo carmine was performed using a sclerotherapy needle to achieve elevation. A mucosal incision was made with a dual knife (Olympus), and the submucosal space was accessed. A long and wide tunnel was created extending proximally from the anal canal. For more efficient injection and dissection, a triangle knife (Olympus) also was used. The electrosurgical unit (ESG-300; Olympus) was set to PulseCut Slow mode (effect 2, 40 W) for both knives. Cut mode was used in areas with low vascularity, and PowerCoag (effect mode, effect 2, 40 W) mode was used in highly vascular regions. A second tunnel was created on the opposite side using the same technique. The tunnels were merged by repositioning the patient according to gravity. Prominent vascular structures encountered during submucosal dissection were coagulated with hemostatic forceps. The lesion was resected en bloc using sponge-holder forceps. The resection area was clean after the procedure, and no muscular injury was observed (Fig. 3A and B, Video 1, available online at www.videogie.org).

**Figure 4:**
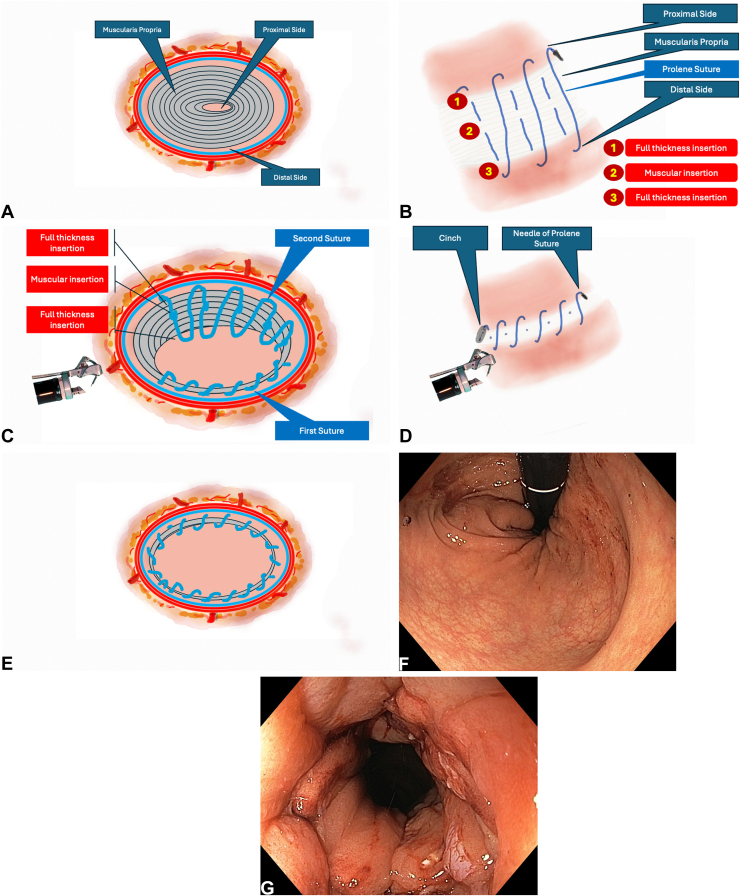
**A,** Schematic view of the resection area. **B,** Schematic view of the suturing strategy. **C,** Schematic view of the suturing strategy. **D,** Schematic view of the postprocedural suturing strategy. **E,** Schematic view of the postprocedural suturing strategy. **F,** Endoscopic view of the resection area in retroflexed position after suturing. **G,** Endoscopic view of the resection area in straight position after suturing.

The resected area was closed using a double-channel endoscope and the endoscopic suturing system with 3 sutures and cinches each. The suturing strategy was as follows: the proximal margin of the defect was grasped and retracted with a tissue grasper to enable a full-thickness suture. The same suture was passed through the muscularis propria in the middle of the defect and completed at the distal margin with another full-thickness insertion. In total, 3 sutures and 3 cinches were used to achieve complete closure. At the end of the procedure, the endoscope passed proximally without resistance (Fig. 4A-G, Video 1).

**Figure 5:**
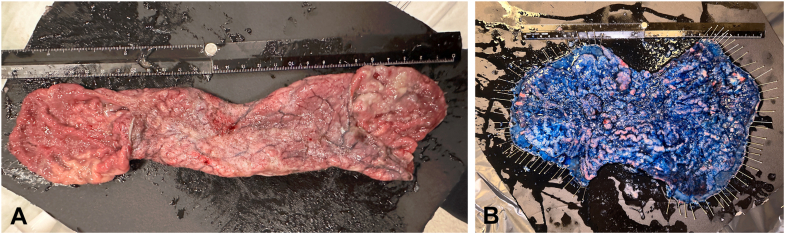
**A,** Macroscopic view of the circumferentially resected specimen. **B,** Macroscopic view of the circumferential resection specimen opened and pinned on a Styrofoam board (total size: 324 × 214 mm).

**Figure 6:**
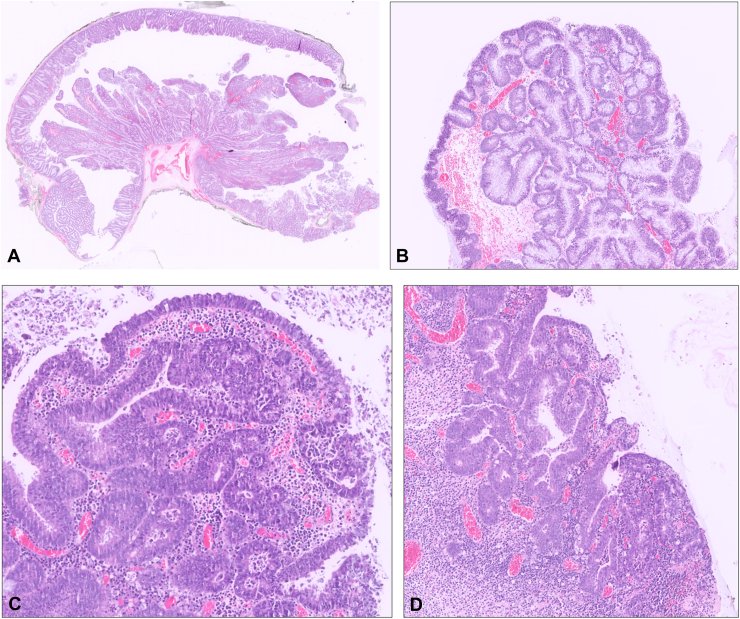
**A,** The lesion had both polypoid and laterally spreading components; hematoxylin & eosin, orig. mag.×0.25. **B,** Both components are composed of adenomatous epithelium; hematoxylin & eosin, orig. mag.×5. **C,** Focal areas of high-grade dysplasia were observed; hematoxylin & eosin, orig. mag. ×10. **D,** Focal areas of high-grade dysplasia were observed; hematoxylin & eosin, orig. mag.×5.

The ESD procedure lasted 229 minutes, with an additional 28 minutes required for suturing, resulting in a total procedure time of 257 minutes. The resected specimen was pinned on a Styrofoam board and measured 324 × 214 mm (Fig. 5A and B, Video 1). Histopathologic examination revealed a tubulovillous adenoma with high-grade dysplasia and negative margins (Fig. 6A-D).

**Figure 7:**
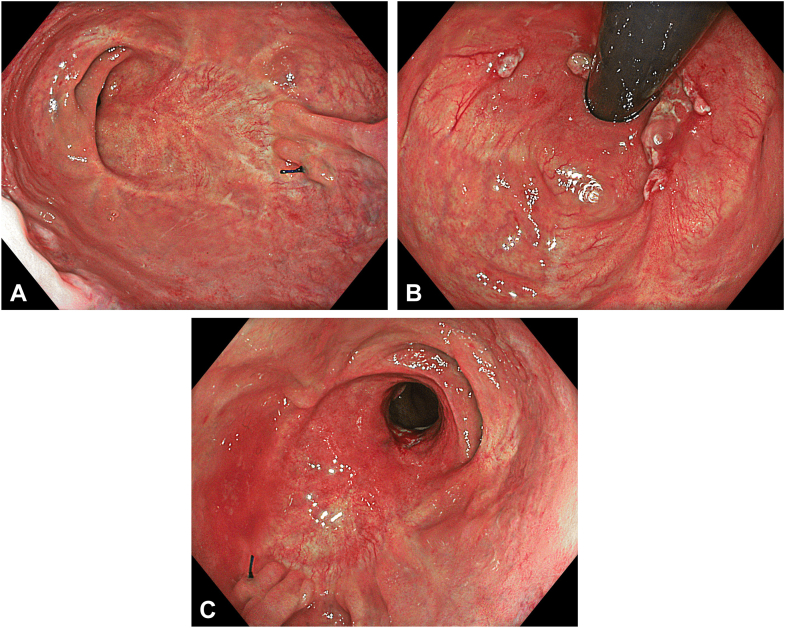
**A,** Endoscopic view of the scar area in straight position 1 year after resection. **B,** Endoscopic view of the scar area in retroflexed position 1 year after resection. **C,** Endoscopic view of the scar area in straight position 1 year after resection.

The patient was admitted to the ward postoperatively. During the first 2 days of follow-up, she experienced a sensation of tenesmus, but no pain, fever, or bleeding was noted. She remained hospitalized for 3 days for observation and completion of parenteral antibiotic therapy and was discharged on postoperative day 3 without any adverse events. On follow-up colonoscopy, the endoscope traversed the anastomotic site without resistance. Although an endoscopically apparent narrowing was observed, it was not clinically significant, and no symptomatic stricture or recurrence was detected during the 24-month follow-up period (Fig. 7A-C, Video 1).

## Discussion

Large LSTs have significant diagnostic and therapeutic challenges. Discordance between biopsy results and final pathology after endoscopic resection is not uncommon.^5^ The risk of malignancy varies according to the type and anatomical location of the LST.^1^ In particular, rectal LSTs greater than 4 cm have a greater incidence of cancer than those in other locations.^2^ In our case, comprehensive radiological and endoscopic evaluations confirmed the lesion's suitability for endoscopic resection.

As an alternative to endoscopic techniques such as EMR or ESD, total mesorectal excision for rectal cancer carries significant perioperative and long-term functional risks. Short-term adverse events include anastomotic leakage (5%-15%), pelvic sepsis, and bleeding, with anastomotic leakage causing the greatest concern because of its association with mortality and permanent stoma formation.^6^^,^^7^ Long-term sequelae significantly impact quality of life, with genitourinary dysfunction affecting 15% to 80% of patients as a result of sexual and urinary dysfunction, including retention and incontinence.^8^^,^^9^ Low anterior resection syndrome, characterized by urgency, frequency, clustering, and fecal incontinence, occurs in 50% to 90% of patients after sphincter-preserving surgery.^10^^,^^11^ These impairments are more severe in patients who receive neoadjuvant radiotherapy and those with low anastomoses, necessitating thorough preoperative counseling and postoperative management.^12^^,^^13^

Although wide-field EMR is a feasible option for such lesions, the recurrence and residual adenoma rates increase significantly for lesions larger than 4 cm.^14^ In such cases, repeat EMR or ESD becomes technically more demanding and increases the risk of adverse events such as perforation.^15^ Therefore, ESD was considered reasonable in this case.

The endoscopic treatment of large LSTs is technically challenging because of their size and anatomical constraints. Factors such as fibrosis, rich vascular networks, peristalsis, difficult angulation, limited luminal width, and thin bowel walls contribute to procedural difficulty.^4^^,^^16^^,^^17^ Lesions larger than 10 cm require significantly prolonged procedure times, increasing the risk of adverse events.^4^^,^^18^^,^^19^ In recent years, techniques such as saline immersion, single or multitunneling, pocket creation, and various traction methods have been developed to enhance dissection quality and safety.^20^, ^21^, ^22^, ^23^

In our case, we used MTT, which we previously described.^24^ This method naturally provides traction by using the scope's tension within the tunnel to expose the submucosal space and facilitate dissection. This approach allows for effective visualization and rapid dissection with minimal injection. Vascular structures are controlled at their source close to the muscle rather than at the branches, improving hemostasis and reducing bleeding risk. By keeping the scope parallel to the muscularis layer, muscle injury and the adverse impact of peristalsis are minimized. Our lesion was prevented from collapsing into the lumen, and dissection orientation was kept. These advantages enabled a safe and rapid en bloc resection.

Common adverse events after ESD-related wide mucosal defects include bleeding, perforation, and long-term strictures.^17^^,^^25^ Circumferential mucosal defects involving more than 90% of the lumen are associated with a significantly increased risk of stenosis,^25^^,^^26^ often requiring additional endoscopic therapy. Endoscopic suturing has been shown to limit neovascularization and fibroblast activity, thereby reducing scar formation and preventing strictures.^27^^,^^28^ In this case, suturing allowed successful closure, enabling early discharge, and prevented stenosis during long-term follow-up.

Incomplete closure, disruption of the closure line, or dead space formation due to mucosa-to-mucosa-only apposition are known risks of endoscopic defect closure.^29^ These issues are theoretically more likely in large mucosal defects. To overcome these challenges and ensure effective closure, several methods including layered closure, mucosa-submucosa closure, and the origami technique have been developed.^30^, ^31^, ^32^ However, these methods may not provide sufficient tissue apposition in the stomach, where the muscularis propria is thicker.^33^ To avoid such problems in our case, nonabsorbable polypropylene sutures were used to achieve full-thickness bites from the proximal edge, through the muscularis propria in the defect center, to the distal margin. This reduced the risk of dead space formation and ensured durable closure. Additionally, only 3 sutures and 3 cinches were used to minimize the risk of luminal narrowing. This strategy also distributed tension evenly across the suture line, reducing the risk of spontaneous dehiscence. No adverse event was observed during follow-up.

In conclusion, ESD is an effective treatment for large LSTs with both diagnostic and curative benefits. The multitunneling technique is a practical, equipment-free approach that facilitates en bloc resection and improves dissection efficiency. Moreover, effective endoscopic suturing enhances procedural safety and optimizes recovery by minimizing both early and delayed adverse events following ESD.

## Patient consent

The patient in this article has given written informed consent to publication of their case details.

## Disclosure

All authors disclosed no financial relationships.

## Supplementary data

Video 1.A >30-cm circumferential rectal laterally spreading tumor treated with multitunnel endoscopic submucosal dissection and suturing (8.45 seconds).
